# Puerperal Albuminuria

**Published:** 1890-10

**Authors:** S. P. Deahofe

**Affiliations:** Potsdam, O.


### Southern Medical Record.

A MONTHLY JOURNAL OF MEDICINE AND SURGERY.

VOL. XX. ATLANTA, GA., OCTOBER, 1890. No. 10.

### Original Articles.

### PUERPERAL ALBUMINURIA.

Read in the Section of Obstetrics and Diseases of Women, at the Forty-first Anual Meeting of the American Medical Association, Nashville, Tenn., May, 1890.

BY S. P. DEAHOFE, M. D., POTSDAM, O.

By albuminuria, I mean a condition in which albumen appears in the urine. It may or it may not be associated with a decided diffuse nephritis ; I say decided, for it is more than likely that if albumen exists for any length of time in the urine, there is more or less nephritis.

I have never been able to distinguish between albumen of Bright's disease and that where the disease does not exist. Semmola, in the *Archives of Physiology*, states that there is a diffrence in the appearance of the precipitate ; and that the albumen of Bright's disease diffuses more rapidly through animal membranes.

A very important form of albuminuria is that found during pregnancy; most frequently in primiparæ, frequently associated with other symptoms of Bright's disease. The following is a very interesting case:

Mrs. D., aged 32, primipara. Family history: Father was an epileptic; other than this it was good. The patient's previous health had been moderately good, she was not robust, but rather poorly developed. Married October 23, 1887, became pregnant about December 24, 1887. On April 28, 1888, she first noticed that her feet were swelling. This swelling rapidly increased until May 13, when I called to see her. Her entire body was œdematous, including hands and face. There was difficulty of breathing, due to œdema of the lungs. Heart was acting fairly well. She complained of headache and nausea, but there were neither vertigo nor any trouble of vision. Bowels were constipated. I ordered the urine saved for twenty-four hours and a specimen brought to my office. Amount passed, 16 ozs.; sp. gr. 1032, albumen in large quantity.

I informed the friends of the gravity of the case and put her upon a skimmed milk diet, and gave her tr. digitalis and bitartrate of potassium to increase the urinary secretion, saline cathartics to act upon the bowels, and vapor baths to act upon the skin. The patient continued to grow worse, and on May 17, the sp. gr. of the urine having reached 1038, albumen increased, the prospects of the case rather gloomy, I called for a consultation. Drs. Baker and Brandon met me next day at 10 a. m., symptoms were still growing worse, amount of urine passed during the previous twenty-four hours, 12 oz., sp. gr. 1040; urine became absolutely thick upon the addition of nitric acid.

The result of the consultation was to continue previous treatment not more than forty-eight hours unless improvement took place; if not, to induce labor.

May 19, condition about the same, except increased headache, and a peculiar heaviness of the tongue was noticed in talking. The lips were now also markedly œdematous.

May 20, 8 a. m. Temperature, which had previously been normal, now rose to 100 deg. The treatment has been faithfully carried out, but the œdema has increased, the urine remains scanty, sp. gr. 1042, albumen very abundant. I made no quantitative analysis, but upon the addition of nitric acid to the urine in a large test-tube it became so thick that it would not run out of the tube when everted.

May 20, 8 a. m., went to the patient for the purpose of inducing labor, but upon arriving at the house, the symptoms seemed slightly better and, hoping that an improvement was about to take place, I postponed the operation to watch the further progress of the case.

May 21. Temperature normal, œdema slightly diminished, sp. gr. 1032, albumen about the same, possibly slightly diminished. Treatment was continued.

May 22, 8 a. m., temperature 99.2 deg., sp. gr. 1030, albumen about the same, treatment continued.

May 23, 9 a. m., temperature normal, sp. gr. 1022, albumen about the same, œdema same. Severe headache, slight nausea, and slight transient dimness of vision complained of by the patient. Treatment continued, except that the cathartics were withdrawn. 4 p. m., severe headache continues, severe nausea and vomiting with pain in the epigastric region now comes on, cannot retain milk. 9 p. m., headache continues unabated, vomiting ceased, dimness of vision now marked, patient says she sees flashes of light before her eyes. Muscular twitchings are now noticed.

10:20 p. m., convulsions. I arrived in a few minutes. The patient had not yet regained consciousness fully. Gave her 1-2 gr. of sulphate of morphia hypodermically, and inhalations of chloroform. Mouth was bleeding from a bite of the tongue during the convulsion. 11 p. m. In the presence of Dr. Brandon I introduced a male elastic catheter into the uterus and let it remain. I found the os low down, soft, and open sufficiently to admit the finger. I stayed with the patient all night. She slept most of the time under the influence of the morphia. Has passed no urine since 1 p. m.

May 24, 9 a. m. Has no pain and expresses herself as feeling quite good. 10 a. m., some bloody discharge from the vagina, but no pains yet. 1:30 p. m., passed about 4 ozs. of urine, the first in twenty-four hours, sweating profusely; occasional pains are now noticed referable to the uterus. Temperature 100.5 deg., sp. gr. 1034, albumen the same. 8:30 p. m., rests quietly, uterine contractions feeble, but increasing. 11 p. m., called and found pains much increased; was delivered of a female fœtus at 10:40 a. m., without an unfavorable symptom, except the temperature, which remained elevated at 100.5 deg.

May 25, 8 a. m., temperature 100 deg., slight headache. 10:30 a. m., sweating freely, headache continues, otherwise feels as well as could be expected. Continued the digitalis, bitartrate of potash, and cathartics. 4:30 p. m., was called on account of patient having difficulty in breathing, and found it due to pulmonary œdema, quite marked upon the right side, evidently due to her lying mostly on that side, the vesicular murmur being almost absent. I changed her position to a sitting posture and to the left side, with relief of the dyspnœa. Urine is much increased, sp. gr. 1020, albumen reduced more than one-half.

From this time on the patient continued to improve. The cathartics were withdrawn and tr. ferri chloridi was added, also lime water was added to the milk on account of some nausea. Dismissed the case June 1.

I might add here that she was again delivered with forceps of a healthy male child May 24, 1889, having passed through her pregnancy without any of her former trouble.

The subject of puerperal albuminuria has attracted the attention of obstetricians for many years, and is well known to be associated in ways still imperfectly understood with many important puerperal diseases. In the severer cases a well-marked parenchymatous nephritis exists; but if every case of albuminuria in pregnancy is due to nepritis, it is probably a form of the disease which may neither lead to severe symptoms nor to chronic disease; however, the absence of severe symptoms or the continuance into the chronic form may often be explained by the most rational of all treatment, viz.: the removal of the cause. On the other hand, the appearance of albumen in the urine of a pregnant woman, though not calling for active interference, should be regarded as a danger signal, and put the physician on the lookout for other indications of renal disease.

There are a number of theories as to its causation, such as a poor quality of blood, an altered condition of the blood, which, on account of the call for nutritive supply on the part of the fœtus, contains an excess of albuminious material. Any condition producing sudden hyperæmia of the kidneys giving rise to a state analogous to the first stage of Bright's disease, such as sudden exposure to cold and impeded cutaneous action.

PRESSURE OF THE GRAVID UTERUS.

Prof. Edes, of Harvard University, thinks that in many instances it is the result of impeded abdominal circulation, although it is rare that the gravid uterus can press upon the renal veins.

Bartels shows that the renal veins occupy a position which secures them from pressure.

Pleyfair says: "The obvious fact that in pregnancy the vessels supplying the kidneys are subjected to mechanical pressure from the gravid uterus, etc., suggests that here we may find an explanation of the frequent occurrence of albuminuria; that this is further strengthened by the fact that the albumen rarely appears until after the fifth month."

That this last theory is not correct is shown by the history of the case just reported, in which the albumen appeared prior to the fourth month, at which time the gravid uterus could not certainly have pressed upon the renal veins.

The symptoms of albuminuria of pregnancy are by no means constant. Anasarca is probably the most frequent symptom which attracts the attention of both patient and physician, not only the swelling of the lower extremities, but the face and upper extremities also

Any puffiness of the face and hands should give rise to suspicion and lead to an examination of the urine. The anasarca may extend throughout the whole body as it did in the case reported, the lungs not escaping. Headache, dizziness, dimness of vision, spots before the eyes, nausea, sleeplessness, irritability of temper, are often met with, and should cause suspicion and lead to an examination of the urine, which is generally scanty and high-colored, and may contain, in addition to the albumen, epithelial cells, tube casts and blood corpuscles. The most formidable of all is eclampsia.

I believe it may be taken as proved that albuminuria is not necessarily accompanied by eclampsia, although the two are almost invariably combined.

Not only is the mother's life in danger ultimately, but there is a strong predisposition to abortion, due to imperfect nutrition of the fœtus by blood impoverished by the drain of albuminous materials through the kidneys. Tanner mentions four cases out of seven which he attended that aborted, one of them three times in succession.

### TREATMENT.

The treatment should consist of such remedies as tend to promote the secretion of the urine and thus relieve, or at least diminish, the congestion of the renal vessels. Saline diuretics, such as the acetate or bitartrate of potash, will best answer this indication. Purgatives which produce watery motions should be administered. Dry cupping over the loins may have a beneficial effect in lessening the renal hyperæmia. The action of the skin should also be promoted by the use of vapor baths. The diet should consist of skimmed milk, with which may be allowed a little whitefish. Tr. of chloride of iron is excellent, and may be combined with tr. of digitalis, which acts as an excellent diuretic.

In obstinate cases, where all these fail, we must consider the advisability of producing premature labor.

### IS THIS OPERATION JUSTIFIABLE ?

Barker says it should be resorted to only after treatment, thoroughly and perseveringly tried, has been without success.

Hofmeier says it does not increase the risk of eclampsia, aud may avert it altogether.

Lusk says as far as his experience goes, the practice of waiting upon Nature has proved uniformly disastrous, while the induction of labor has furnished recoveries.

Braun says he has known but one patient to recover between the fourth and six months of pregnancy except where abortion has taken place.

I believe it is perfectly justifiable in all cases attended with symptoms of great gravity; and I should not hesitate to adopt this resource in cases in which the quantity of albumen is great and progressingly increasing, and in which treatment has been of no avail, and especially if attended with such symptoms as severe headache, dizziness and loss of sight.

As it is not likely to be indicated until the child has reached a viable age, although in my case it will be noticed that it was much earlier, and the child's life is in danger from albuminuria, we are justified in performing the operation for the mother's safety alone.

THE MEDICO-LEGAL ASPECT.

This involves a question of menico-legal importance. Charges of inadequacy of cause for its performance may be made, and the physician should take all precaution to avoid these, and thus save him the unpleasantness of a prosecution for a criminal offence.

Tidy says (Vol. iii, page 100): "It is manifest that if any question should arise or action at law be commenced against a medical man for inducing abortion, it would be necessary to show: 1, that there was a necessity for the operation, the life of the mother being at stake, and the operation being less to be feared than natural delivery; and 2, that the action was *bona fide*."

Now, it may be difficult for a practitioner to successfully defend himself against a charge of inadequacy of cause for its performance, owing to the increasing number of conditions which justify the induction of premature labor; therefore we urge upon medical men, 1, not to induce premature labor or abortion without the most mature deliberation; 2, not to undertake it until after consultation with a second practitioner; 3, in any case to have the full consent of the husband or guardian, in writing, if possible.

Taylor says (p. 592): "This practice has been condemned as immoral and illegal, but it is impossible to admit that there can be any immorality in performing an operation to give a chance of saving the life of a woman when, by neglecting to perform it, it is almost certain that both herself and the child will perish.

"Any question respecting its illegality cannot be entertained, for the means are administered or applied with the *bona fide* hope of benefiting the female, and not with any criminal design."

When eclampsia supervenes what shall we do? Shall we attend to the convulsions and let the labor take care of itself, as Gooch says? There seems to be a difference of opinion upon the subject, one advising the immediate emptying of the uterus, others who leave the labor entirely alone. Evidently that course which seems least likely to prove a source of irritation to the mother is the one adopted, and the practitioner must exercise his own judgment as to what course he will pursue. But when convulsions come on during the earlier months of pregnancy, as they did in my case, there seems, to my mind at least, to be no doubt as to the course to pursue, viz.: to bring on labor with the least possible irritation to the mother. The introduction into the uterus, without rupturing the membranes, of a flxeble male catheter, seems to be the best way.

To control the convulsions there are several remedies recommended. If the woman be a strong, healthy one, with marked evidences of great cerebral congestion, and vascular tension, such as a full, bounding pulse and a livid face, venesection is a valuable remedy. Of the sedatives which are useful, chloroform probably stands at the head of the list. Hydrate of chloral, per rectum, or in combination with bromide potash per os, is highly recommended. That which served me best was hypodermic injections of morphia in half grain doses, together with inhalations of chloroform intermittently.—

American Medical Journal.

